# A deep-red fluorophore based on naphthothiadiazole as emitter with hybridized local and charge transfer and ambipolar transporting properties for electroluminescent devices

**DOI:** 10.3762/bjoc.19.122

**Published:** 2023-11-03

**Authors:** Suangsiri Arunlimsawat, Patteera Funchien, Pongsakorn Chasing, Atthapon Saenubol, Taweesak Sudyoadsuk, Vinich Promarak

**Affiliations:** 1 Department of Materials Science and Engineering, School of Molecular Science and Engineering, Vidyasirimedhi Institute of Science and Technology, Wangchan, Rayong 21210, Thailand; 2 Frontier Research Center, Vidyasirimedhi Institute of Science and Technology, Wangchan, Rayong 21210, Thailand

**Keywords:** ambipolar material, deep red fluorophore, hybridized local and charge transfer, naphthothiadiazole, OLED, organic light-emitting diode

## Abstract

Herein, we report the synthesis and characterization of an efficient ambipolar charge-carrier-transporting deep-red fluorophore (**TPECNz**) based on a donor–acceptor–donor (D–A–D)-type molecule and its application as a non-doped emitter in an organic light-emitting diode (OLED). The fluorophore **TPECNz** contains naphtho[2,3-*c*][1,2,5]thiadiazole (Nz) as a strong acceptor unit symmetrically functionalized with *N*-(4-(1,2,2-triphenylvinyl)phenyl)carbazole as a donor and aggregation-induced emission (AIE) luminogen. The experimental (solvatochromic and emission in THF/water mixtures studies) and theoretical investigations prove that **TPECNz** retains cooperative hybridized local and charge transfer (HLCT) and weak AIE features. Thanks to its D–A–D-type structure with a proper twist angle between the D and A units, a strong electron deficiency of the Nz unit, and electron-donating and hole-transporting natures of carbazole, **TPECNz** exhibits a strong deep red emission (λ_em_ = 648 nm) with a high fluorescence quantum yield of 96%, outstanding thermal property (*T*_g_ = 236 °C), and ambipolar charge-carrier-transporting property with a decent balance of mobility of electrons (1.50 × 10^−5^ cm^2^ V^−1^ s^−1^) and holes (4.42 × 10^−6^ cm^2^ V^−1^ s^−1^). **TPECNz** is successfully employed as a non-doped emitter in an OLED which displays deep red electroluminescent emission peaked at 659 nm with CIE coordinates of (0.664, 0.335)), an EQE_max_ of 3.32% and exciton utilization efficiency (EUE) of 47%.

## Introduction

Recently, organic fluorophores with efficient deep-red/near-infrared (DR/NIR) emission properties (λ_em_ = 650–900 nm) have received much attention due to their potential applications in several different fields such as chemosensing [1–3], bioimaging/biosensing [4–8], photodynamic therapy [9], optical communication [10], NLO materials [11], laser dyes [12], and DR/NIR electroluminescent devices [13–18]. However, DR/NIR chromophores typically suffer from low photoluminescent quantum yields (PLQY) because of their intrinsic small band-gap energy causing larger vibronic coupling between the ground and excited states, particularly when they are applied as emitters in organic light-emitting diodes (OLEDs) [19]. Therefore, with such a constrained number of efficient emitters, the current advance of DR/NIR OLEDs largely trails behind the visible light emission OLEDs [20–21]. So far, remarkable efforts have been made in designing new DR/NIR organic fluorophores in combination with device engineering to build up the performance of DR/NIR OLEDs. In general, to realize high fluorescence in the DR/NIR region from the organic fluorophores, the challenges of their pronounced non-radiative processes, and high planarity and extended π-conjugation lengths which eventually favor the undesired formation of poorly emissive molecular aggregates must be overcome. Alternatively, donor–acceptor (D–A)-type organic fluorophores have been introduced and successfully exploited for the development of fluorophores with DR/NIR emissions without lengthy extension of their π-conjugation systems due to a broadening of both the valence and the conduction bands and a consequent narrowing of the energy gap [22–25]. The D–A characters also offer a tunability of optoelectronic properties such as energy levels, optical bandgap (*E*_g_), and charge-transport properties, which could be done by selecting different D and A moieties [22,26–28]. In these fluorophores, the emission generally derives from intramolecular charge-transfer (CT) states at lower energy. Nonetheless, the formed CT state between D and A in such materials normally caused a lesser Φ_PL_ because of the separated frontier molecular orbitals [29–30]. Recently, the reports by Ma et al. revealed that by designing suitable twist-angles between D and A and D–A strength, frontier molecular orbitals of D–A fluorophores are not completely separated due to overlap between the transition orbitals [31–33]. In these molecules, the lowest excited state still shows moderate or large oscillator strengths or a mixing of two excited-state components, locally excited (LE) state and CT excited state. This kind of excited state was later known as a hybridized local and charge transfer (HLCT) state, in which the CT component can provide a high-lying reversed intersystem crossing (hRISC) pathway for fast and effective triplet utilization in the device, and the LE component can contribute good PLQY [34–36]. So far, based on this concept, several D–A-type fluorophores have been designed and investigated including some DR/NIR materials [37–41]. Inevitably, these materials displayed high PLQY and exceptional OLED device performance [42–45]. Even though the radiative transition rate is improved for the HLCT fluorophores, the non-radiative transition process is still an obstacle to lowering the PLQY, particularly for the DR/NIR emitters [20,46]. Therefore, to enhance the performance of HLCT-based DR/NIR fluorophores the non-radiative transition process of the exited state needs to be resolved. This could be managed by the molecular design of the D–A structure. It has been reported that the performance of HLCT fluorophores greatly depends on the strength of donors, acceptors, and π spacers and the building blocks of HLCT should be carefully selected to regulate appropriate electron push–pull strength [32,35,47–48].

**Scheme 1:**
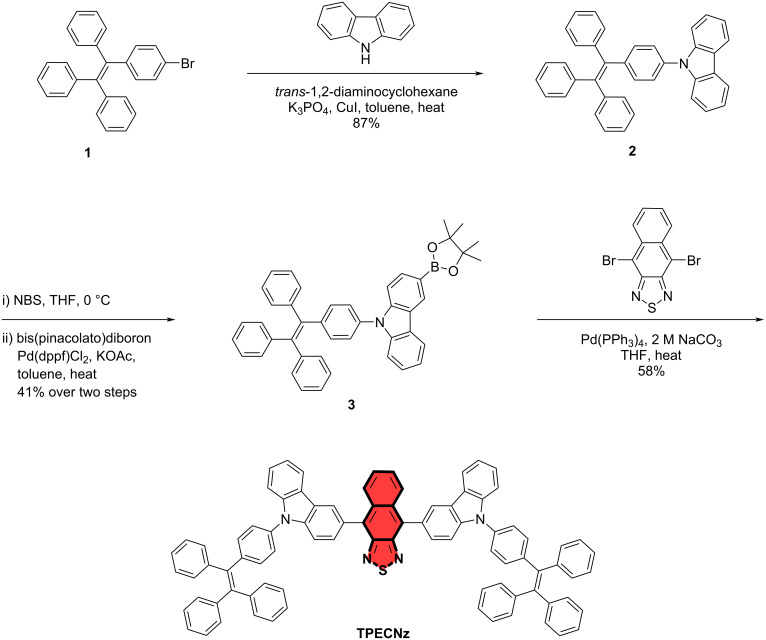
Synthesis of D–A–D chromophore **TPECNz**.

Here, a novel chromophore, 4,9-bis(9-(4-(1,2,2-triphenylvinyl)phenyl)-9*H*-carbazol-3-yl)naphtho[2,3-*c*][1,2,5]thiadiazole (**TPECNz**), built on a D–A–D type structure was designed and synthesized (Scheme 1). The D–A–D configuration was constructed by incorporating a second symmetrical donor in the D–A framework, which could further reduce the energy gap between HOMO/LUMO hybrid orbits and drive fluorescence emission to longer wavelengths [48–49]. In this molecular design, the strong electron-deficient naphtho[2,3-*c*][1,2,5]thiadiazole (Nz) [13,50–51] as an acceptor and the strong electron-donating carbazole [52] as a donor unit were used in the D–A–D structure to further reduce the bandgap, thereby red-shifting the absorption and emission wavelengths. In addition, the strong electron affinity of Nz and the electron-donating and hole-transporting ability of carbazole would build in the good ability to transport electrons and holes, respectively, providing well-balanced ambipolar characteristics. On the other hand, the attached 4-(1,2,2-triphenylvinyl)phenyl (TPE) moiety would induce aggregation-induced emission (AIE) as feature into the molecule. It was suspected that AIE would restrain the aggregation-caused quench (ACQ) and the energy loss through the vibronic coupling natures of the DR/NIR fluorophores [53]. As a result, **TPECNz** effectively possesses a strong deep-red emission with combined HLCT, weak AIE, and ambipolar transporting properties, which enabled its application as non-doped emitter. The **TPECNz**-based non-doped deep-red OLED realized a maximum external quantum efficiency (EQE_max_) of 3.32% with an emission peak at 659 nm.

## Results and Discussion

The designed **TPECNz** molecule was synthesized by a multistep reaction as described in Scheme 1. Initially, an Ullmann coupling reaction of bromo-TPE **1** and carbazole provided TPE-*N*-carbazole **2** in good yield (87%). Compound **2** was then converted to the boronic ester intermediate **3** in 41% yield over two steps: monobromination at the carbazole unit of **2** with NBS/THF at low temperature giving the unisolated mixed brominated product followed by borylation with bis(pinacolato)diboron catalyzed by Pd(dpf)Cl_2_/KOAc. Finally, **TPECNz** was obtained as red solid in a reasonable yield by a Suzuki-type cross-coupling reaction between **3** and 4,9-dibromonaphtho[2,3-*c*][1,2,5]thiadiazole. The chemical structure and purity of compound **3** were verified by ^1^H NMR, ^13^C NMR, and high-resolution MALDI-TOF-MS techniques.

**Figure 1:**
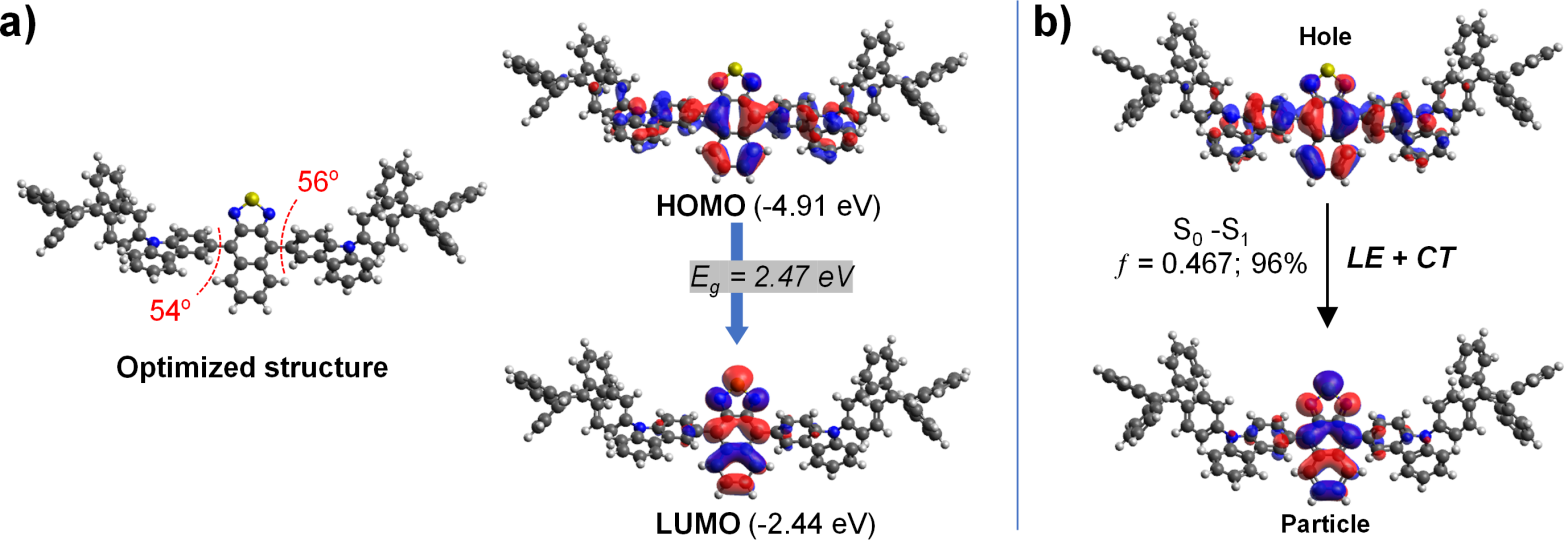
a) The optimized structure and HOMO/LUMO distributions calculated by B3LYP/6-31G(d,p) method. b) The natural transition orbits (NTOs) of the S_0_ → S_1_ transition computed by CAM-B3LYP/6-31G(d) method. The percentage is the proportion of transition and ƒ is the oscillator strength.

To examine the electronic properties of D–A **TPECNz**, density functional theory (DFT) calculations at the B3LYP level of theory with the 6-31G(d,p) basis set were performed. It has been previously reported that the twist angle of the D–A segment has a significant role in controlling the CT component in the HLCT state [54]. A suitable twisted angle (40–80°) allocated an appropriate tuning between the complete π‐conjugation and the pure CT transition character to form the HLCT state. As depicted in Figure 1a, the optimized structure of **TPECNz** revealed a twisted molecular configuration with dihedral angles of 54–56° between the planes of the terminal carbazole donors and the Nz acceptor that are beneficial for the state mixing between the LE and CT states. The lowest unoccupied molecular orbital (LUMO) was mainly localized on the Nz ring, while the highest occupied molecular orbital (HOMO) was delocalized over the Nz core and the attached carbazole moieties. There was an overlap between the HOMO and LUMO orbitals over the Nz fragment, which is likely to promote the LE component and consequently gives rise to a faster radiative decay and thus a higher luminescence efficiency. To further figure out excited-state properties of **TPECNz**, the natural transition orbitals (NTOs) of singlet (S) and triplet (T) excited states were executed based on time-dependent (TD)-DFT calculations at the CAM-B3LYP/6-31G(d) level of theory. As shown in Figure 1b, the hole and particle of **TPECNz** are similar to its HOMO and LUMO orbitals, respectively. The S_0_ → S_1_ transition with oscillator strength (ƒ) of 0.476 clearly showed HLCT transition characteristics, in which a certain overlap between hole and particle wavefunctions on the Nz ring represented for LE component inducing a high luminescence efficiency, while a significant spatial separation between hole and particle wavefunctions considered for CT component promoting RISC process along high-lying excitation state for enhancing an exciton utilization efficiency (EUE).

**Figure 2:**
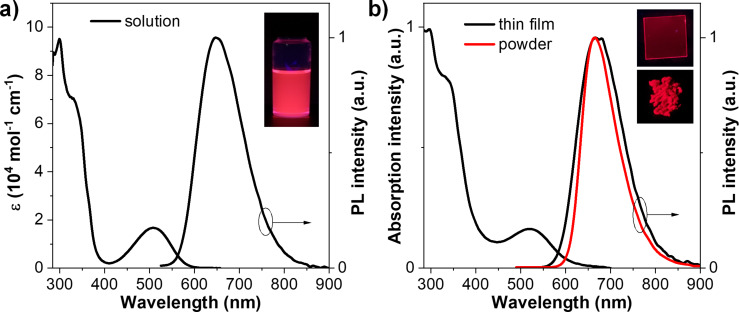
UV–vis absorption and PL spectra in a) toluene solution (≈1 × 10^−5^ M) and b) thin film spin-coated on fused silica substrate and solid powder (insert: fluorescence images of the solution, thin film, and solid powder under UV light at 356 nm).

The photophysical properties of **TPECNz** were studied in solution, thin film, and solid powder. As shown in Figure 2a, the UV–vis absorption spectrum in diluted toluene solution displays intense absorption peaks in the high energy region (<380 nm) and a much weaker absorption peak at 508 nm attributed to the π–π* transition of the conjugated aromatic backbone and intramolecular charge-transfer (ICT) transition from carbazole donor to Nz acceptor, respectively. Such weak ICT absorption peak (ε = 17,000 M^−1^ cm^−1^) as compared to the π–π* absorption peak (ε = 95,300 M^−1^ cm^−1^) symbolizes a weak electronic coupling between the carbazole donor and the Nz acceptor parts because of the twisted configuration between them as observed in the optimized structure (Figure 1a) [47]. In solution, **TPECNz** exhibits a strong deep-red emission with the PL spectrum (λ_em_ = 648 nm) described by a broad PL band (FWHM = 120 nm) that reflects the corresponding ICT absorption band well, featuring no significant vibronic structure, and a considerably large Stokes shift of 140 nm. The UV–vis absorption and PL spectra of spin-coated films were similar to those of dilute solutions. Based on the onset energy of this UV–vis spectrum, the optical band gap (*E*_g_^opt^) was estimated to be 2.04 eV. **TPECNz** in thin film and solid powder also displayed deep-red emission observed by PL maxima at 668 and 665 nm, respectively (Figure 2b). As listed in Table 1, the **TPECNz** solution shows an excellent absolute PL quantum yield (PLQY) of 96%, whereas the spin-coated thin film exhibits a red-shifted spectrum with the PLQY dropped to 35% due to intermolecular interactions. However, the emission efficiency of the molecule is enhanced in the solid powder form with a PLQY of 64% owing to its AIE nature.

**Table 1:** Optical and physical data of **TPECNz**.

λ_PL_ (nm) sol^a^/film^b^/solid^c^	τ (ns)^d^ sol^a^/film^b^	PLQY (%)^e^ sol^a^/film^b^/solid^c^	*k*_r_/*k*_nr_ (10^7^ s^−1^)^a^	*k*_r_/*k*_nr_ (10^7^ s^−1^)^b^	*E*_1/2_ vs Ag/Ag^+^ (V)^f^	*E*_g_^opt^/*E*_g_^ele^ (eV)^g^	HOMO/LUMO (eV)^h^

648/668/664	13.4/11.1	96/35/64	7.16/0.30	3.15/5.86	−1.26, 1.05, 1.47, 1.72	2.04/2.17	−5.44/−3.40

^a^Measured in diluted toluene solution (1 × 10^−5^ M); ^b^measured in thin film; ^c^measured in solid powder; ^d^transient PL decay lifetime with excitation at 475 nm; ^e^absolute PL quantum yield measured by an integrating sphere; ^f^obtained from differential pulse voltammetry (DPV) peak; ^g^band-gap energy from UV absorption (*E*_g_^opt^) and electrochemical (*E*_g_^ele^) results; ^h^calculated from HOMO (eV) = −(4.44 + *E*_onset_^ox^) and LUMO (eV) = HOMO + *E*_g_^opt^.

**Figure 3:**
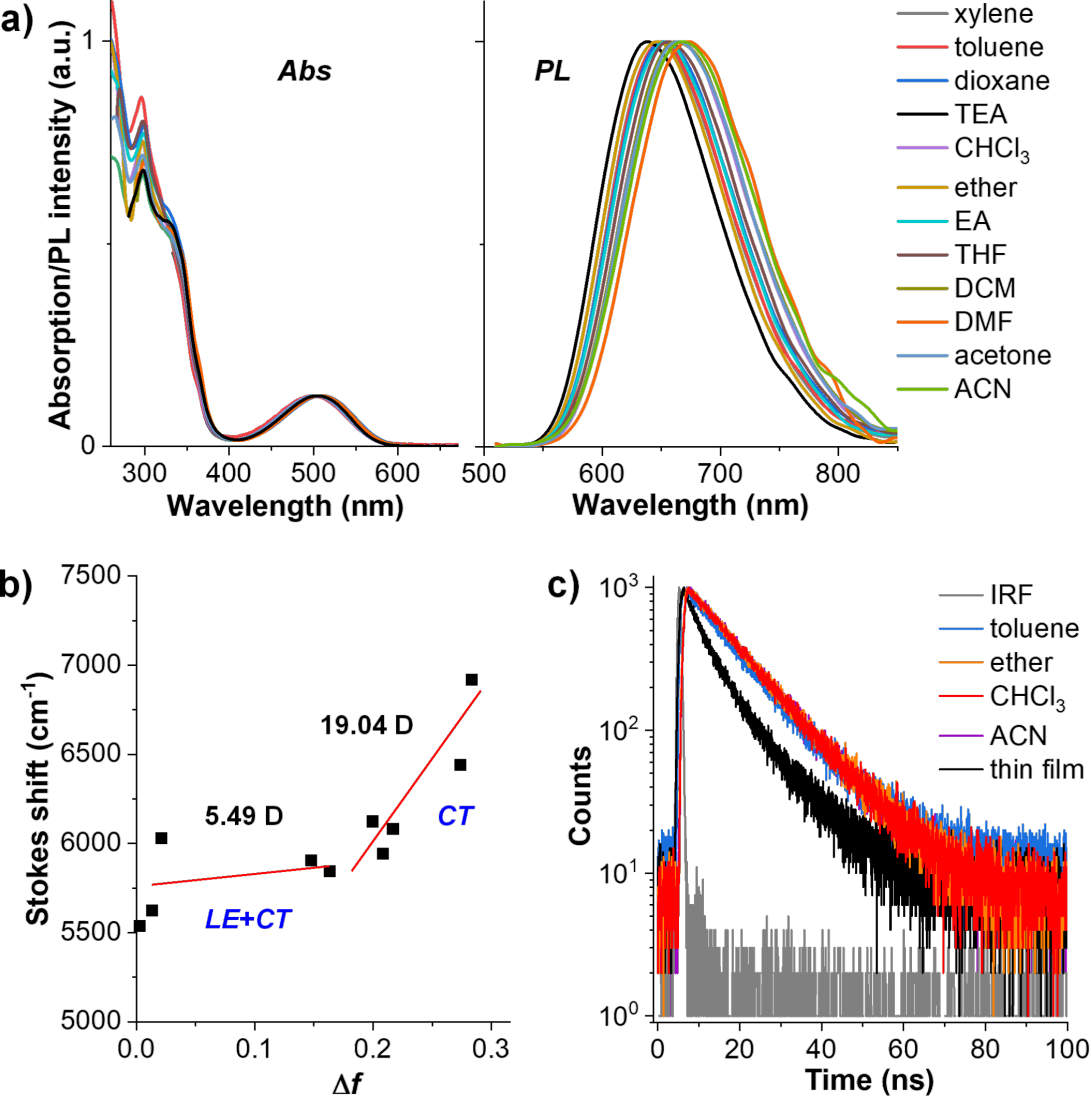
a) Normalized UV–vis absorption/PL spectra in different solvents. b) Lippert–Mataga plot of Stokes shift vs solvent polarity function (Δ*f*). c) Transient PL decay traces in various solvents and thin film. IRF = instrument response function.

To gain more information about the effect of the solvent on the optical properties of **TPECNz**, the absorption and emission behaviors were investigated in several solvents as illustrated in Figure 3a. The results showed that, while the UV–vis absorption spectra were nearly unaffected by the nature of the solvent due to the LE nature, the emission spectra exhibited a noticeable positive solvatochromism, with maxima shifting towards longer wavelengths with increasing solvent polarities. Particularly, the maximum PL peaks in low polar solvents showed little shifts because of the LE character, whereas red shifts of the PL maxima apparently occurred in higher polar solvents due to CT excited state; therefore, the molecule contains both the intrinsic LE and CT excited states or demonstrates HLCT characteristics. Besides, the Stokes shifts between absorption and emission spectra were plotted as a function of solvent polarity function (Δ*f*) corresponding to the Lipper–Mataga model which defines the interactions between the solvent and dipole moment of the chromophore (Figure 3b). The Lippert–Mataga plot showed two linear slopes indicating the presence of two different excited states in the molecule. In the high-polarity region, the excited-state dipole moment (μ_e_) was 19.04 D, which was close to that of a typical CT molecule 4-(*N*,*N*-dimethylamino)benzonitrile (μ_e_ = 23 D) [55], suggesting a CT state-dominated character in high-polarity solvents. Besides, in the low-polarity region, the μ_e_ value was 5.49 D, which is slightly higher than the ground-state dipole moment (μ_g_ = 1.30 D) estimated using B3LYP/6-31G(d,p) calculation. Nevertheless, this value was significantly smaller than that of the high-polarity region, indicating that the S_1_ state in low-polarity solvents contained both CT and LE components simultaneously, which underwent interstate coupling forming a new HLCT emissive state. Additionally, transient PL decay spectra of **TPECNz** in solvents of different polarities showed a single exponential decay in nanosecond ranges (Figure 3c), signifying that the excited state responsible for the PL emission originates from the hybridization between LE and CT excited states or HLCT state, not a simple mix-up of the two states [56].

Furthermore, to further understand the PLQY of the molecule in solution and thin film, the radiative rate constant (*k*_r_) and non-radiative rate constant (*k*_nr_) were calculated from the PLQY values and PL lifetimes (τ) according to Equation 1 and Equation 2.


[1] $$k_{\text{r}}=\frac{\Phi_{\text{PL}}}{\tau }$$

[2] $$\Phi_{\text{PL}}=\frac{k_{\text{r}}}{k_{\text{r}}+k_{\text{nr}}}$$

As can be seen from Table 1, the *k*_r_ of **TPECNz** in solution is larger than *k*_nr_, which is consistent with the the observerd high PLQY in solution. On the other hand, *k*_nr_ of **TPECNz** in thin films is significantly higher than the *k*_nr_ of solution, explaining the drop of PLQY in thin films as compared to the PLQY in solution. However, the moderate PLQY in thin films still makes **TPECNz** suitable for the deep-red OLEDs.

**Figure 4:**
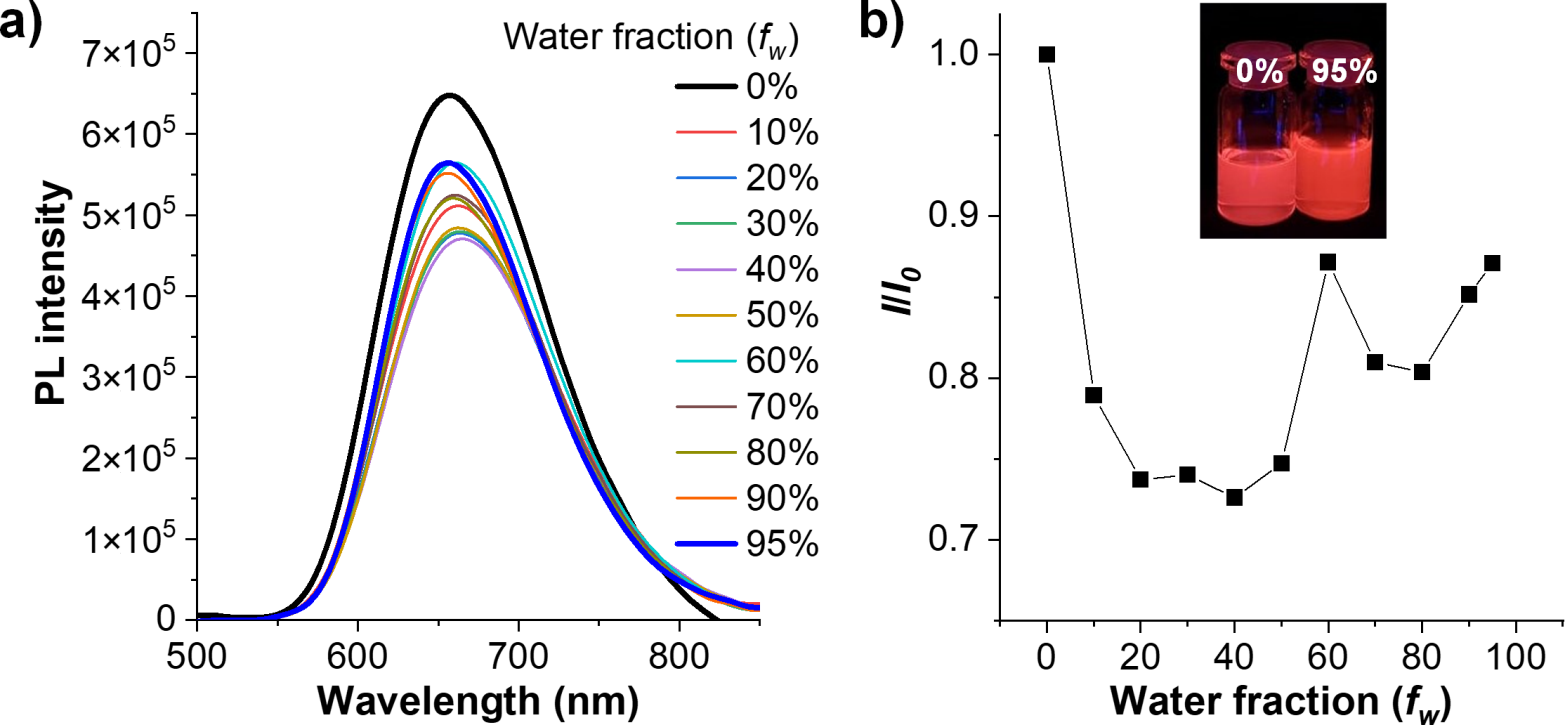
a) PL spectra in THF/water mixtures (5 μM) with different water fractions (*f*_w_). b) Plot of relative PL intensity (*I*/*I*_0_) vs % of water fraction (*f*_w_) (insert: photographs of the solutions @ *f_w_* of 0% and 95% taken under UV illumination).

In addition, the AIE characteristic of **TPECNz** was further investigated by observing its PL emissions in water/THF mixtures with different water fractions (*f_w_* = 0–95%). In such diverse solvent mixtures, the molecule will demonstrate different degrees of aggregation since it can dissolve really well in THF but is insoluble in water, resulting in colloidal nanoaggregates being formed in solutions with high water contents. As shown in Figure 4a and 4b, in pure THF, **TPECNz** deep-red emission color with the PL peaked at 658 nm. With the increase of *f_w_*, the PL emission maxima suddenly dropped. At *f_w_* = 40%, the PL intensity reached the lowest point, as well as the PL peak being red-shifted. This could be attributed to the twisted ICT emission characteristics of the molecule described by a red-shifted PL emission and a weakened emission intensity with the increased solvent polarity [57–58]. As the *f_w_* was further raised to 95%, the PL emissions became more intense and were blue-shifted. This was attributable to the formation of nanoaggregates and suppression of the ICT process. This finding proves that both **TPECNz** is an AIE-active fluorophore, however, the AIE effect in **TPECNz** might not be as strong as for the standard AIE molecules, whose emissions are completely quenched in solutions and brightened by aggregate formation, as a result of the restriction of intramolecular motions (RIM) caused by intermolecular steric interaction [53]. The weak AIE characteristic of **TPECNz** in the aggregate state could be attributed to the competition between the twisted ICT and AIE properties in the molecule [57–58].

**Figure 5:**
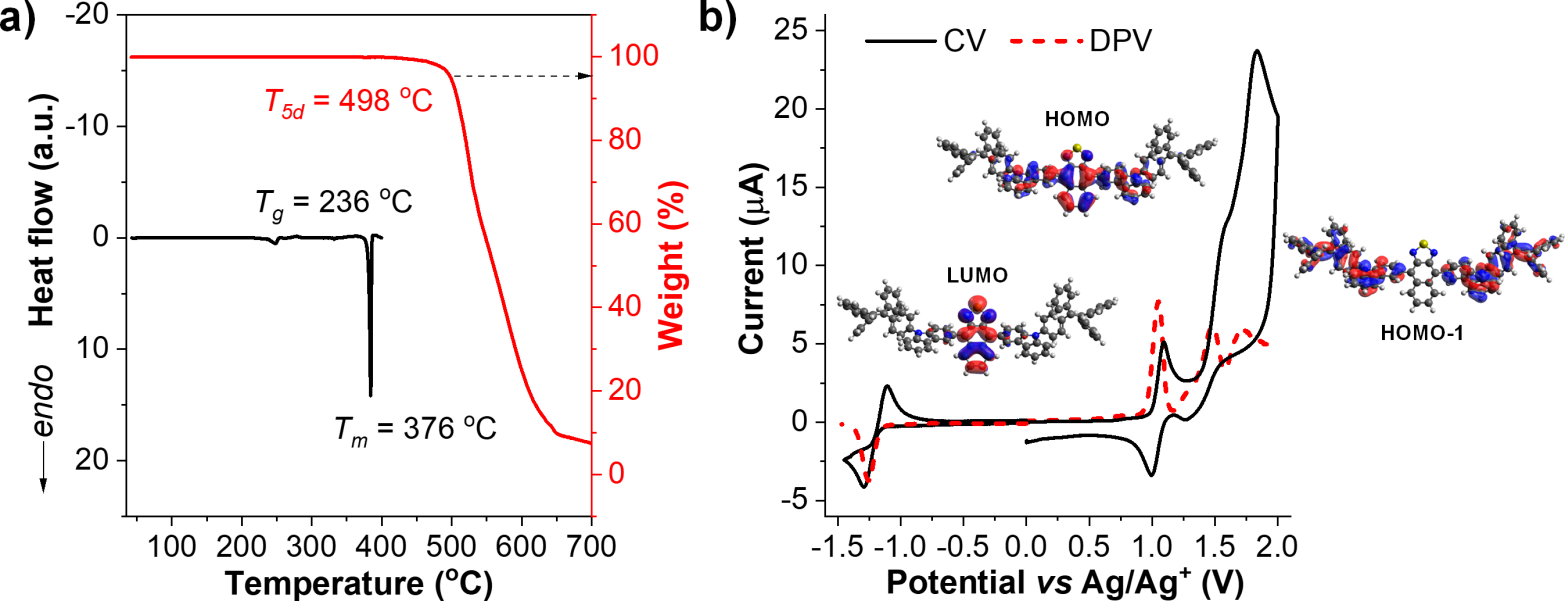
a) DSC and TGA thermograms measured at a heating rate of 10 °C min^−1^ under N_2_ flow. b) Cyclic voltammogram (CV) and differential pulse voltammogram (DPV) analyzed in dry CH_2_Cl_2_ at a scan rate of 50 mV s^−1^ under an argon atmosphere (insert: HOMO/HOMO−1/LUMO orbitals).

The thermal properties of **TPECNz** were studied by thermogravimetric analysis (TGA) and differential scanning calorimetry (DSC) under N_2_ atmosphere at a scanning rate of 10 °C min^−1^. As displayed in Figure 5a, the compound has a high thermal stability with a decomposition temperature at 5% weight loss (*T*_5d_) of 498 °C, a glass transition temperature (*T*_g_) of 236 °C, and a melting temperature (*T*_m_) of 376 °C. The high *T*_g_ may enhance the morphological thin film stability and film integrity of **TPECNz** during device fabrication. While the high *T*_m_ suggests that **TPECNz** can withstand the latent heat generated during the device fabrication and operation. These results signaled that **TPECNz** may be suitable for optoelectronic devices. The electrochemical behavior of **TPECNz** was analyzed by cyclic voltammetry (CV) and differential pulse voltammetry (DPV) in CH_2_Cl_2_ containing 0.1 M *n-*Bu_4_NPF_6_ as a supporting electrolyte. As illustrated in Figure 5b, the molecule shows multiple quasi-reversible oxidation and reduction behavior in the potential window from −1.5 eV to 2.0 eV. The reduction wave appeared at a half-wave potential (*E*_1/2_) of −1.26 eV assigned to the reduction of an electron-deficient Nz core as observed in the calculated LUMO orbital [39]. The first oxidation wave occurred at *E*_1/2_ of 1.05 eV and was ascribed to the oxidation of the π-conjugated backbone along the Nz core and end-capped carbazole moieties as seen in the computed HOMO orbital, while the second oxidation wave at *E*_1/2_ of 1.47 eV was attributed to the oxidation of the π-conjugated TPE–carbazole fragment as depicted in the calculated HOMO−1 orbital. In addition, the oxidation and reduction onsets of **TPECNz** were 1.00 eV and −1.17 eV, respectively. Hence, the electrochemical energy gap (*E*_g_^ele^) defined as the difference between the oxidation and reduction onset potentials was calculated to be 2.17 eV, which is slightly higher than the *E*_g_^opt^ estimated from the UV–vis absorption onset (Table 1). The HOMO energy level was calculated from the oxidation onset potential to be −5.44 eV. Further, the calculated LUMO energy level was found to be −3.40 eV. The relatively low LUMO level of **TPECNz** is conceivably associated with a high electron deficiency of the Nz moiety originating from its localized distribution of electrons and the high electronegativities of the N and S atoms, whereas its high HOMO level is attributed to the electron-donating property of the attached carbazoles and the π-conjugation of the carbazole–Nz–carbazole fragment. Such proper energy levels will benefit the efficient charge injections from the electrodes in OLEDs.

**Figure 6:**
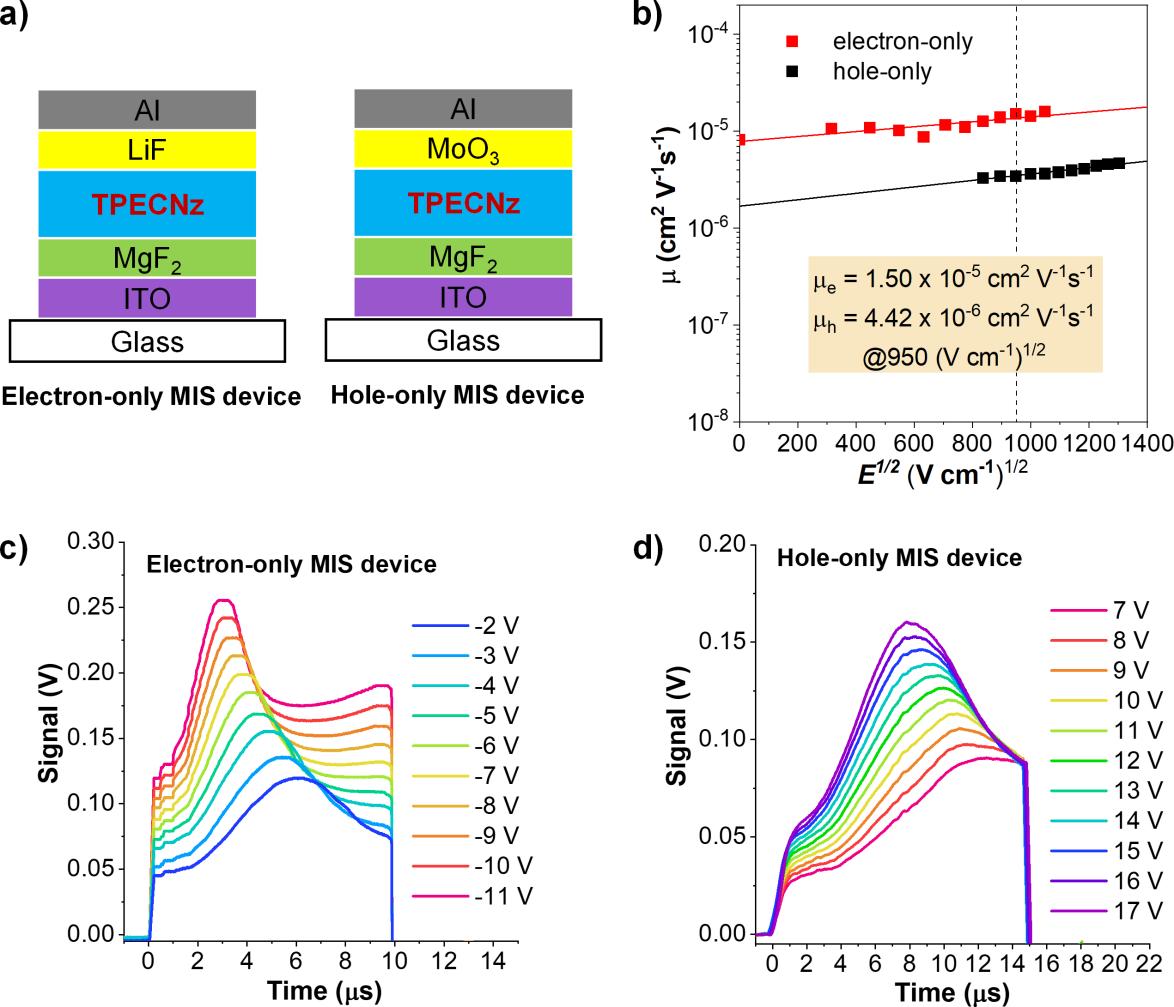
a) Schematic structure of the hole-only and electron-only MIS devices. b) Electric-ﬁeld dependence of the hole and electron mobilities. Transient signals at different applied maximum voltages for c) electron-only MIS device and d) hole-only MIS device.

To evaluate charge-carrier mobility in **TPECNz**, hole (μ_h_) and electron (μ_e_) mobilities were initially measured using metal-insulator-semiconductor (MIS) diodes in combination with charge extraction in linearly increasing voltage (CELIV) or MIS-CELIV technique [59–61]. Electron- and hole-only MIS devices were fabricated with the structures of indium tin oxide (ITO)/magnesium fluoride (MgF_2_) (20 nm)/**TPECNz** (100 nm)/lithium fluoride (LiF) (1 nm)/aluminum (Al) (100 nm) and ITO/MgF_2_ (20 nm)/**TPECNz** (100 nm)/molybdenum trioxide (MoO_3_) (6 nm)/Al (100 nm), respectively (Figure 6a), where the MgF_2_ layer was employed as an insulator. During the measurements, varied maximum voltages were applied during the pulse for extracting the charges while keeping the pulse duration and oﬀset voltage of 10 μs and 5 V for the electron-only MIS device and 15 μs and −5 V for the hole-only MIS device, respectively, and their MIS-CELIV signal transient plots as a function of time are shown in Figure 6c and 6d. As the applied voltage increased, it was found that the transient peak shifted to slightly shorter times indicating an increasing carrier mobility. The hole and electron mobilities (μ) were calculated and plotted as a function of electric ﬁeld (*E*^1/2^). As illustrated in Figure 6b, the mobility (μ) of both holes and electrons is electric field dependent and gradually increases on increasing the electric ﬁeld, which is explained as the Poole–Frenkel effect obeying the relationship, log µ ∝ *E*^1/2^ [59]. The measured mobilities of the holes (μ_h_) and electrons (μ_e_) of **TPECNz** thin film at 950 (V cm^−1^)^1/2^ electric field were 4.42 × 10^−6^ and 1.50 × 10^−5^ cm^2^ V^−1^ s^−1^, respectively. This result suggested that **TPECNz** was an ambipolar material with the mobility of electrons somewhat greater than that of holes. The pronounced ability of **TPECNz** to transport electrons could be accredited to a strong-electron affinity of the Nz core, while its ability to transport holes could be derived from the attached carbazole moieties.

**Figure 7:**
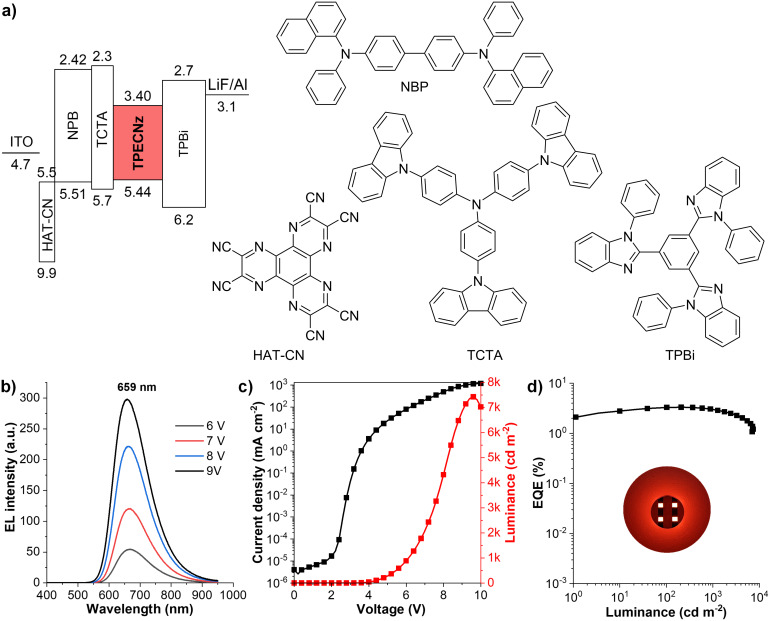
a) Schematic energy diagram of OLED and organic materials used in the device. b) EL spectra at various applied voltages. c) Current density–voltage–luminance (*J*–*V–L*) characteristics. d) External quantum efficiency–luminance (EQE*–L*) plot (insert: photograph of OLED under operation).

To evaluate the electroluminescent (EL) performance of **TPECNz**, non-doped OLED employing **TPECNz** as an emissive layer (EML) was fabricated through thermal evaporation of the optimized device configuration of ITO/1,4,5,8,9,11-hexaazatriphenylene-hexacarbonitrile (HAT-CN) (6 nm)/*N*,*N*'-bis(naphthalen-1-yl)-*N*,*N*'-bis(phenyl)benzidine (NPB) (30 nm)/tris(4-carbazoyl-9-ylphenyl)amine (TCTA) (10 nm)/**TPECNZ** (60 nm)/1,3,5-tris(1-phenyl-1*H*-benzimidazol-2-yl)benzene (TPBi) (40 nm)/LiF (1 nm)/Al (100 nm), in which ITO and Al served as anode and cathode, respectively (Figure 7a). Herein, HAT-CN and LiF were used as the hole- and electron-injection layers, respectively, NPB and TPBi were applied as the hole- and electron-transporting layers, respectively, and TCTA owing to its high-lying LUMO energy level (LUMO = 2.3 eV) and low electron mobility was utilized as an electron-blocking layer [62]. Owing to a narrow band gap stemming from its strong D–A characteristic, **TPECNz**-based non-doped OLED successfully displayed deep-red emission with a maximum EL peak at 659 nm and Commission Internationale de L’Eclairage (CIE) coordinates of (0.664, 0.335), which were very close to the standard red CIE coordinates of (0.67, 0.33) [63]. The device demonstrated high emission stability with EL spectra under different operating voltages (6–9 V) revealing an unchanged profile with a single emission band (Figure 7b), indicating the recombination zone of the excitons confined inside the EML. No emission peaks at low wavelengths from the supporting layers (NPB at 440 nm [64], TCTA at 410 nm [65], and TPBi at 390 nm [66]) and at longer wavelengths due to the excimer/exciplex emissions at the interfaces of NPB-TCTA/EML and EML/TPBi were observed, signifying that the OLED possessed a well-balanced electron and hole transport. Moreover, due to the efficient ambipolar charge-carrier-transporting property of **TPECNz** EML and good charge balance in the device, the non-doped OLED was turned on at a low voltage of 3.2 V and achieved decent device EL performance as presented in Figure 7c and 7d. The device exhibited a maximum luminance (*L*_max_) of 7430 cd m^−2^, a maximum external quantum efficiency (EQE_max_) of 3.32%, and a maximum luminance efficiency (LE_max_) of 2.87 cd A^−1^, with a little efficiency roll-off of 9% at 1000 cd m^−2^. Furthermore, its exciton utilization efficiency (EUE) was calculated using EUE = EQE/(η_out_ × η_rec_ × PLQY) (light outcoupling efficiency: η_out_ = 20%, PLQY in a thin film = 35%, and charge recombination: η_rec_ = 100%) to be 47%. This EUE was higher than the 25% theoretical upper limit of spin statistics for typical fluorescent emitters, indicating that the triplet excitons have been utilized via an HLCT mechanism to contribute to the EL in this device.

## Conclusion

In summary, a new D–A–D-type fluorophore as a deep-red emitter has been designed and synthesized by exploiting a strong electron-accepting naphtho[2,3-*c*][1,2,5]thiadiazole (Nz) as A unit and *N*-(4-(1,2,2-triphenylvinyl)phenyl)carbazole as D unit. The integration of 4-(1,2,2-triphenylvinyl)phenyl (TPE) moiety as an AIE luminogen helped to provide the fluorophore with AIE feature, while its D–A–D structure with a proper twist angle (54–56°) between the Nz acceptor and carbazole donor promotes a state mixing between close-lying LE and CT states or HLCT property. The combined characteristics of HLCT and weak AIE were clearly evidenced by a solvatochromic study, emission in THF/water investigation, and theoretical calculations. These synergetic properties could benefit for effective utilization of excitons in the OLED. A collaborative consequence of its D–A–D-type structure, a strong electron affinity of Nz unit, and electron donating and hole-transporting properties of carbazole nurtured the molecule to exhibit an intense deep-red emission with decent PL quantum yield, superior thermal property, and ambipolar charge-carrier-transporting property with a good balance of mobility of electrons and holes, which were desirable properties for OLED application. It was efficiently utilized as a non-doped emitter in OLEDs. The device presented a strong deep-red electroluminescent (EL) emission (peak at 659 nm and CIE coordinates of (0.664, 0.335)) with a maximum luminance of 7430 cd m^−2^, EQE_max_ of 3.32%, and EUE of 47%. This work successfully established that an ambipolar D–A–D-type fluorophore having combined AIE–HLCT features could be a promising design to develop future deep-red or near-infrared OLEDs.

## Experimental

### Materials and Methods

All chemical reagents and solvents were purchased from commercial resources and used without further purification. ^1^H NMR and ^13^C NMR spectra were recorded with a Bruker AVANCE III HD 600 (600 MHz for ^1^H and 151 MHz for ^13^C) using CDCl_3_ as a solvent containing TMS as an internal standard. High-resolution mass spectrometry (HRMS) analysis was performed using either a Bruker LC-Quadrupole-Time-of Flight tandem mass spectrometer or a Bruker Autoflex MALDI-TOF mass spectrometer. UV–vis spectra were recorded using a Perkin Elmer Lambda 1050 UV–vis–NIR spectrometer. Luminescence emission spectra and lifetimes were analyzed using an Edinburgh Instruments FLS980 Spectrometer. Absolute PL quantum yield (PLQY) was measured using a calibrated integrating sphere incorporated with Edinburgh Instruments FLS980 Spectrometer. Electrochemical studies were carried out using an Autolab PGSTA101 potentiostat equipped with three electrodes (Pt, glassy carbon, and Ag/AgCl) in dry CH_2_Cl_2_ containing 0.1 M *n*-Bu_4_NPF_6_ as a supporting electrolyte under argon. Thermogravimetric analysis (TGA) and differential scanning calorimetry (DSC) were recorded using a Rigaku STA8122 thermogravimetric analyzer and a PerkinElmer DSC 8500 Lab System, respectively. Melting points were measured using a Krüss KSP1N melting point meter and are uncorrected.

Quantum chemical calculations were executed using the Gaussian 16 package [67]. Density functional theory (DFT) calculations at the B3LYP level of theory with the 6-31G(d,p) basis set were performed to realize the ground state geometry, HOMO and LUMO distributions, and HOMO and LUMO energy levels. Natural transition orbitals (NTOs) were calculated for the excited states using time-dependent (TD)-DFT calculations at the CAM-B3LYP/6-31G(d) level of theory.

### Device fabrication and testing

The patterned ITO-coated glass substrate with a sheet resistance of 12 Ω sq^−1^ was pre-cleaned carefully and cured with UV/O_3_ for 20 min. The OLED with an active diode area of 0.04 cm^−2^ was fabricated using a Kurt J. Lasker mini SPECTROS 100 thin film deposition system under vacuum conditions with a base pressure lower than 1 × 10^−5^ bar and a thermal evaporation rate of 0.2–0.3 Å s^−1^ for MgF_2_, HATCN, NPB, TCTA, **TPECNz**, and TPBi, 0.05–0.1 Å s^−1^ for LiF, and about 1 Å s^−1^ for Al layer. The thickness of each layer was monitored by a quartz oscillator thickness sensor. All devices were measured without encapsulation under an ambient atmosphere at room temperature. They were analyzed by a Keithley 2400 source meter, a Hamamatsu Photonics PMA-12 multichannel analyzer, and an integrating sphere equipped with a Hamamatsu Photonics C9920-12 EQE measurement system. The hole and electron mobility were investigated using MIS-CELIV measurement. A pulse delay generator (Stanford Research System DG535) and a single channel arbitrary/function generator (AFG 3021B) were used to generate the CELIV triangle pulse with adjustable voltage slope and offset, as well as amplified the signal by WMA-320 high voltages amplifier. For photo-CELIV, a delay generator (Stanford Research System DG535) was also used for pulse synchronization along with an LED driver (THORLABS, LEDD1B). The signal was recorded by a 3-series mixed domain oscilloscope (Tektronix, MDO32).

**Synthesis of 9-(4-(1,2,2-triphenylvinyl)phenyl)-9*H*-carbazole (2):** [68] A mixture of **1** (2.00 g, 4.86 mmol), carbazole (2.44 g, 14.58 mmol), K_3_PO_4_ (5.17 g, 24.313 mmol), ±-*trans*-1,2-diaminocyclohexane (1.46 mL, 12.17 mmol) and CuI (1.85 g, 9.72 mmol) in dry toluene (50 mL) was degassed with N_2_ for 10 min. The reaction mixture was stirred at reflux under N_2_ atmosphere for 18 h. After being cooled to room temperature, the mixture was diluted with water (100 mL) and extracted with CH_2_Cl_2_ (50 mL × 3). The combined organic layer was washed with water, brine solution, dried over anhydrous Na_2_SO_4_, filtered, and concentrated under reduced pressure. The crude product was then purified by column chromatography over silica gel eluting with CH_2_Cl_2_/hexane 1:4 to give white solids (2.11 g, 87%). ^1^H NMR (600 MHz, CDCl_3_) δ 8.12 (d, *J =* 7.7 Hz, 2H), 7.40 (t, *J =* 7.8 Hz, 2H), 7.34 (d, *J =* 8.2 Hz, 2H), 7.28 (t, *J =* 8.0 Hz, 4H), 7.24 (d, *J =* 8.1 Hz, 2H), 7.21–7.07 (m, 15H); ^13^C NMR (151 MHz, CDCl_3_) δ 143.55, 143.41, 143.30, 142.94, 141.84, 140.74, 140.12, 135.68, 132.67, 131.42, 131.37, 131.32, 127.85, 127.77, 127.72, 126.73, 126.69, 126.62, 126.12, 125.83, 123.32, 120.25, 119.84, 109.80; HRMS–APCI–TOF (*m/z*): [M + H]^+^ calcd for C_38_H_28_N, 498.2216; found, 498.2233.

**Synthesis of 3-(4,4,5,5-tetramethyl-1,3,2-dioxaborolan-2-yl)-9-(4-(1,2,2-triphenylvinyl)phenyl)-9*H*-carbazole (3):** A round-bottomed flask containing a solution of compound **2** (1.81 g, 3.63 mmol) in THF (200 mL) was covered with aluminum foil and cooled in an ice bath. A solution of NBS (646 mg, 3.63 mmol) in THF (100 mL) was added dropwise into the solution over a period of 2 h, while monitoring the reaction by TLC (CH_2_Cl_2_/hexane 1:4). Then, the reaction was quenched with water (100 mL) and the mixture extracted with CH_2_Cl_2_ (50 mL × 3). The combined organic layer was washed with water, brine, dried over anhydrous Na_2_SO_4_, filtered, and concentrated under reduced pressure. The residue was crystallized from CH_2_Cl_2_/methanol to obtain compound **3** as a mixture with unreacted starting material and dibrominated product which was used in the next step without further purification. White solid (1.34 g, 2.32 mmol). HRMS–APCI–TOF *m/z*: [M + H]^+^ calcd for C_38_H_28_N, 498.2216; found, 498.2217; [M + H]^+^ calcd for C_38_H_27_BrN, 576.1321; found, 576.1331; [M + H]^+^ calcd for C_38_H_26_Br_2_N, 654.0427; found, 654.0438.

The mixture of the above crude product (1.00 g), bis(pinacolato)diboron (881 mg, 3.47 mmol), KOAc (2.04 g, 20.81 mmol), and Pd(dppf)Cl_2_ (70.8 mg, 0.087 mmol) in dry toluene (50 mL) was degassed with N_2_ for 10 min. The reaction mixture was stirred at a refluxing temperature under N_2_ atmosphere for 48 h. After being cooled to room temperature, the mixture was diluted with water (100 mL) and extracted with CH_2_Cl_2_ (50 mL × 3). The combined organic layer was washed with water, brine, dried over anhydrous Na_2_SO_4_, filtered, and concentrated under reduced pressure. The crude product was then purified by column chromatography over silica gel eluting with CH_2_Cl_2_/hexane 3:7 to give compound **3** as white solid (928 mg, 41% over two steps). ^1^H NMR (600 MHz, CDCl_3_) δ 8.61 (s, 1H), 8.15 (d, *J =* 7.7 Hz, 1H), 7.85 (d, *J =* 8.2 Hz, 1H), 7.39 (t, *J =* 7.7 Hz, 1H), 7.34–7.22 (m, 7H), 7.22–7.04 (m, 15H), 1.40 (s, 12H); ^13^C NMR (151 MHz, CDCl_3_) δ 143.54, 143.38, 143.24, 143.13, 142.88, 141.95, 140.91, 140.13, 135.48, 132.70, 132.35, 131.43, 131.38, 131.32, 127.88, 127.77, 127.73, 127.70, 126.75, 126.72, 126.65, 126.15, 125.86, 123.54, 123.06, 120.50, 120.25, 109.83, 109.19, 83.66, 24.95; HRMS–APCI–TOF (*m/z*): [M + H]^+^ calcd for C_44_H_39_BNO_2_, 624.3068; found, 624.3078.

**Synthesis of 4,9-bis(9-(4-(1,2,2-triphenylvinyl)phenyl)-9*H*-carbazol-3-yl)naphtho[2,3-*c*][1,2,5]thiadiazole (TPECNz):** A mixture of **3** (834 mg, 1.34 mmol), 4,9-dibromonaphtho[2,3-*c*][1,2,5]thiadiazole (200 mg, 0.58 mmol), Pd(PPh_3_)_4_ (80.6 mg, 0.070 mmol) and 2 M Na_2_CO_3_ (8.7 mL, 17.44 mmol) in THF (50 mL) was degassed with N_2_ for 3 min. The reaction mixture was stirred at refluxing temperature under N_2_ atmosphere for 48 h. After being cooled to room temperature, the mixture was diluted with water (100 mL) and extracted with CH_2_Cl_2_ (50 mL × 3). The combined organic layer was washed with water, brine, dried over anhydrous Na_2_SO_4_, filtered, and concentrated under reduced pressure. The crude product was then purified by column chromatography over silica gel eluting with CH_2_Cl_2_/hexane 3:7, followed by recrystallization from a CH_2_Cl_2_/methanol mixture to obtain **TPECNz** as red solid (396.0 mg, 58%). Mp > 360 °C; ^1^H NMR (600 MHz, CDCl_3_) δ 8.43 (s, 2H), 8.15 (td, *J =* 7.1, 3.5 Hz, 4H), 7.74 (dd, *J =* 8.3, 1.7 Hz, 2H), 7.60 (d, *J =* 8.3 Hz, 2H), 7.45 (t, *J =* 7.6 Hz, 2H), 7.41 (t, 6H), 7.35 (dd, *J =* 7.2, 3.2 Hz, 2H), 7.30 (d, *J =* 8.3 Hz, 6H), 7.24–7.07 (m, 30H); ^13^C NMR (151 MHz, CDCl_3_) δ 152.04, 143.54, 143.40, 143.29, 143.19, 141.93, 141.27, 140.62, 140.10, 135.60, 132.78, 132.46, 131.43, 131.38, 131.31, 130.75, 129.14, 128.16, 127.87, 127.81, 127.72, 127.43, 126.78, 126.71, 126.63, 126.22, 126.17, 123.56, 123.35, 123.21, 120.53, 120.11, 110.03, 109.89; HMRS–MALDI–TOF (*m/z*): [M]^+^ calcd for C_86_H_56_N_4_S, 1176.4226; found, 1176.4221.

## Supporting Information

File 1 Energy diagram of singlet and triplet excited states estimated by CAM-B3LYP/6-31G(d,p) calculations and copies of ^1^H NMR, ^13^C NMR, and HRMS spectra of the synthesized compounds.
