# Supplementary material for: A deep-red fluorophore based on naphthothiadiazole as emitter with hybridized local and charge transfer and ambipolar transporting properties for electroluminescent devices
Source: Beilstein J Org Chem. 2023 Nov 3;19:1664–76. doi: 10.3762/bjoc.19.122 (PMC10630680; doi:10.3762/bjoc.19.122)
Supplement: File 1 — Energy diagram of singlet and triplet excited states estimated by CAM-B3LYP/6-31G(d,p) calculations and copies of 1H NMR, 13C NMR, and HRMS spectra of the synthesized compounds. [file Beilstein_J_Org_Chem-19-1664-s001.pdf]

BEILSTEIN JOURNAL OF ORGANIC CHEMISTRY

Supporting Information

for

A deep-red fluorophore based on naphthothiadiazole as emitter with hybridized local and charge transfer and ambipolar transporting properties for electroluminescent devices

Suangsiri Arunlimsawat, Patteera Funchien, Pongsakorn Chasing, Atthapon Saenubol, Taweesak Sudyoadsuk and Vinich Promarak

Beilstein J. Org. Chem. **2023,** *19,* 1664–1676. doi:10.3762/bjoc.19.122

Energy diagram of singlet and triplet excited states estimated by CAM-B3LYP/6-31G(d,p) calculations and copies of ^{1}H NMR, ^{13}C NMR, and HRMS spectra of the synthesized compounds

License and Terms: This is a supporting information file under the terms of the Creative Commons Attribution License (https://creativecommons.org/licenses/by/4.0). Please note that the reuse, redistribution and reproduction in particular requires that the author(s) and source are credited and that individual graphics may be subject to special legal provisions.
The license is subject to the *Beilstein Journal of Organic Chemistry* terms and conditions: (https://www.beilstein-journals.org/bjoc/terms)

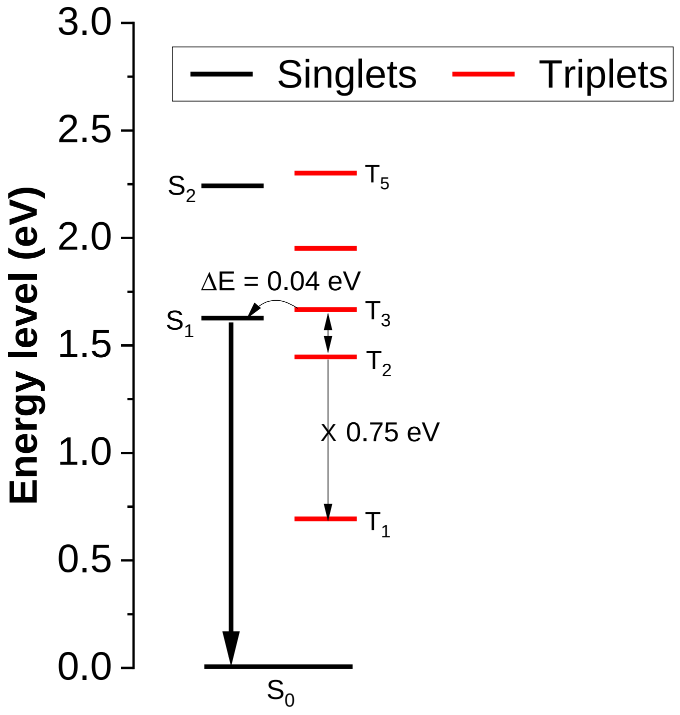

Figure S1: The energy diagram of singlet and triplet excited states of the compound estimated by CAM-B3LYP/6-31G(d,p) calculations.

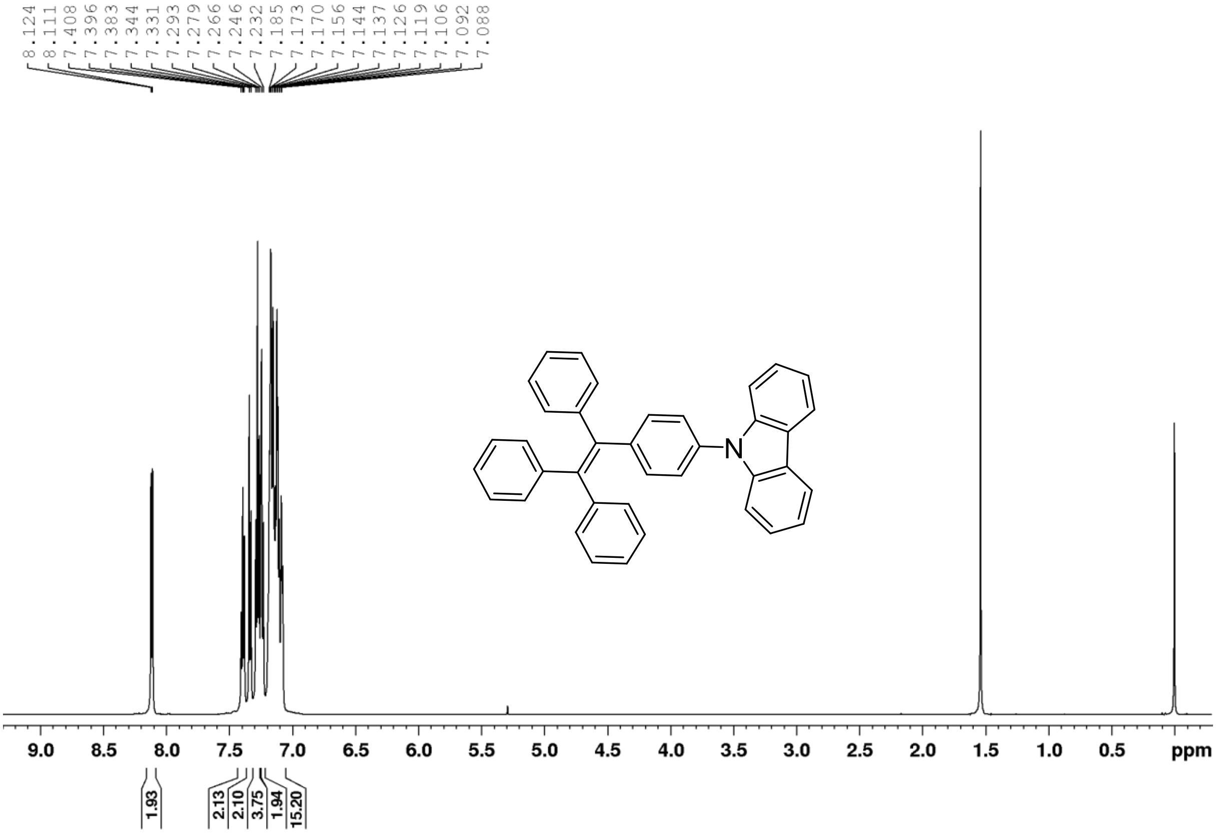

Figure S2: ^{1}H NMR spectrum of **2** in $CDCl_3$ (600 MHz) @ 25 °C.

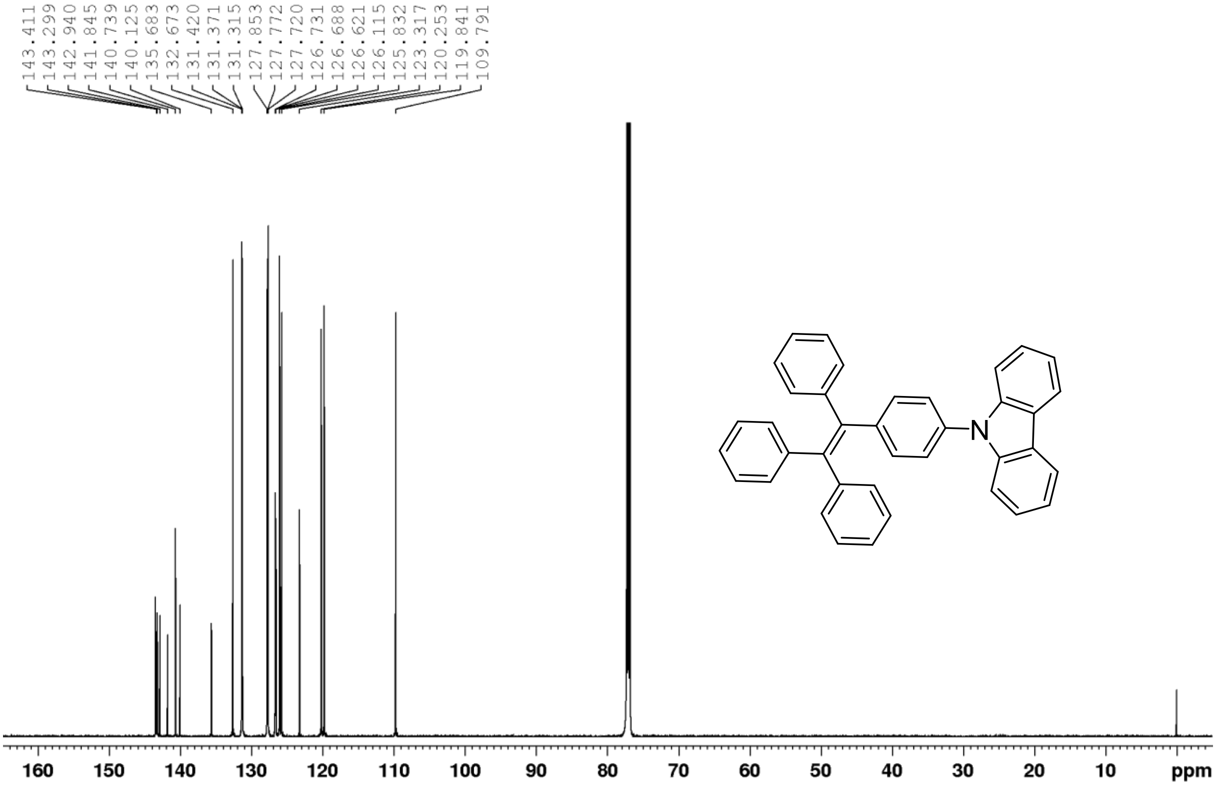

Figure S3: ^{13}C NMR spectrum of **2** in $CDCl_3$ (151 MHz) @ 25 °C.

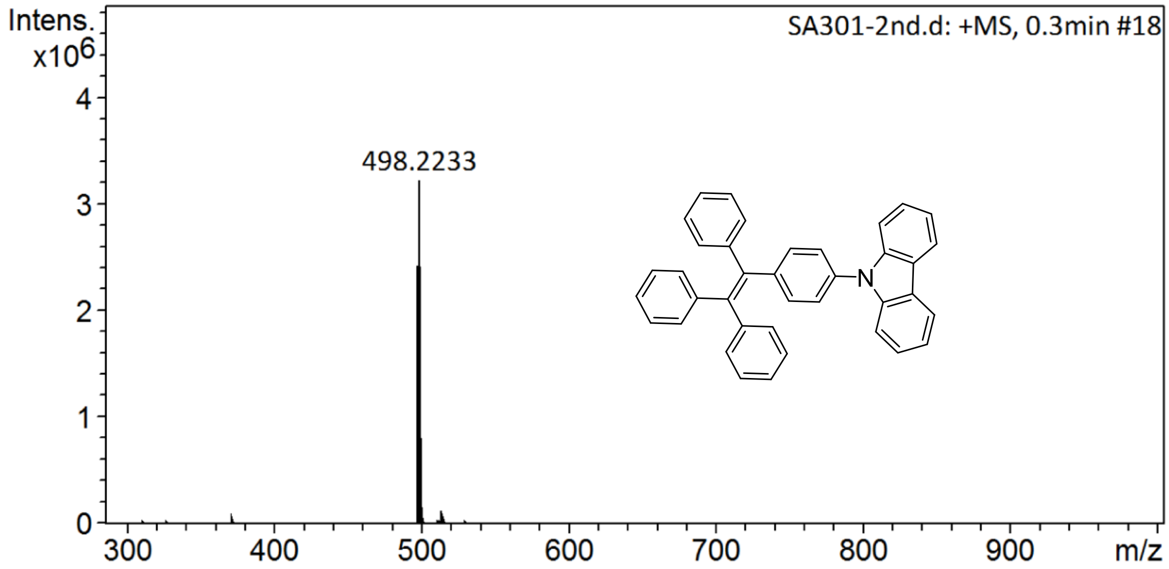

Figure S4: APCI–TOF mass spectrum $[M + H]^+$ of **2**.

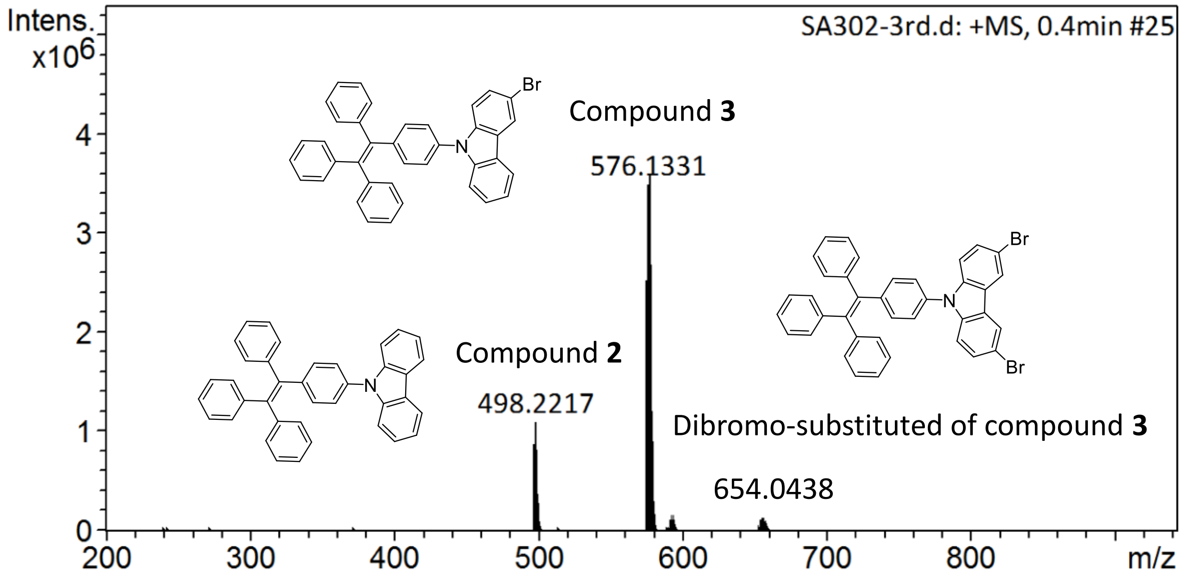

Figure S5: APCI–TOF mass spectrum $[M + H]^+$ of the crude product mixutre from the NBS bromination in the synthesis of compound **3**.

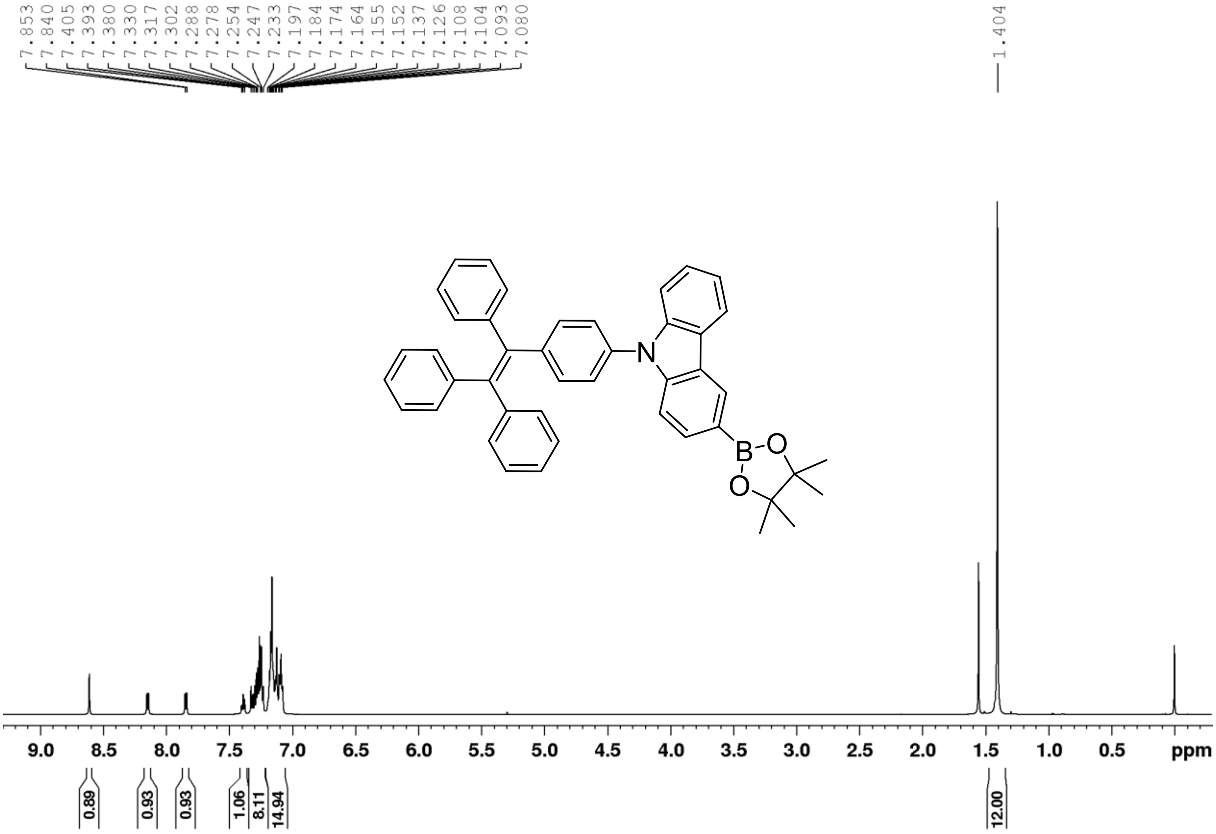

Figure S6: ^{1}H NMR spectrum of **3** in $CDCl_3$ (600 MHz) @ 25 °C.

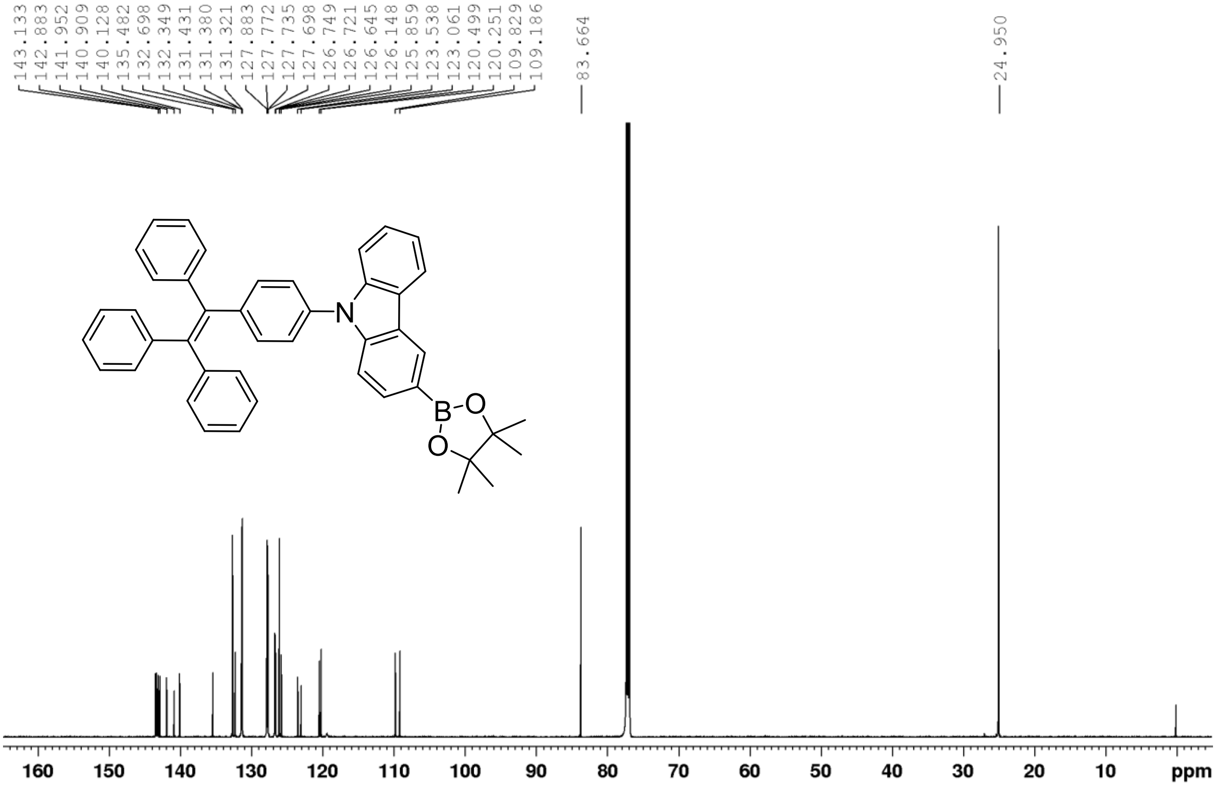

Figure S7: ^{13}C NMR spectrum of **3** in $CDCl_3$ (151 MHz) @ 25 °C.

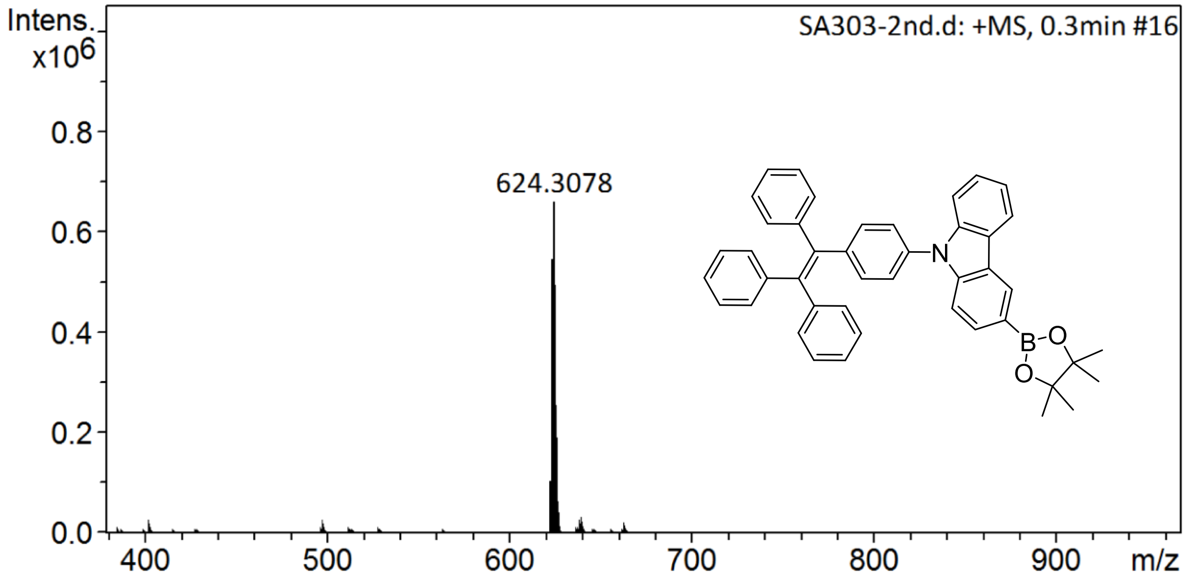

Figure S8: APCI–TOF mass spectrum $[M + H]^+$ of **3**.

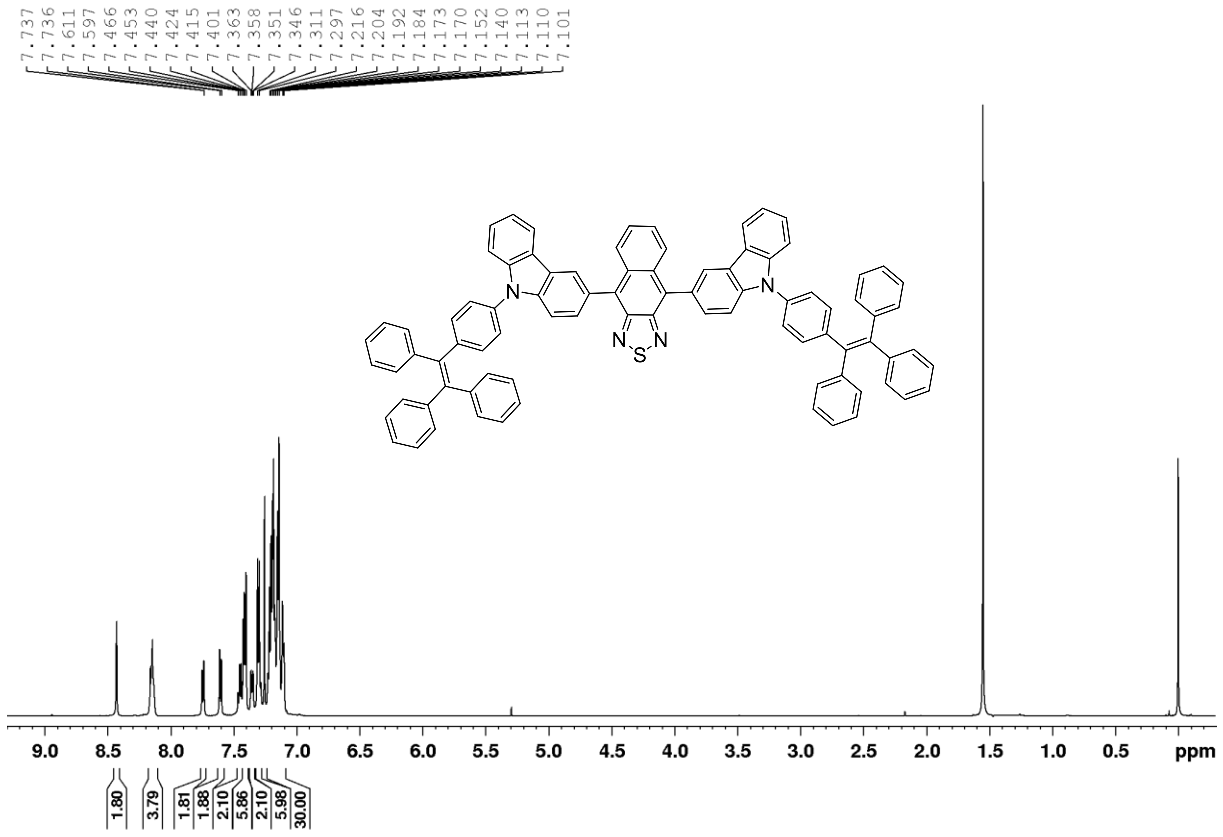

Figure S9: ^{1}H NMR spectrum of **TPECNz** in $CDCl_3$ (600 MHz) @ 25 °C.

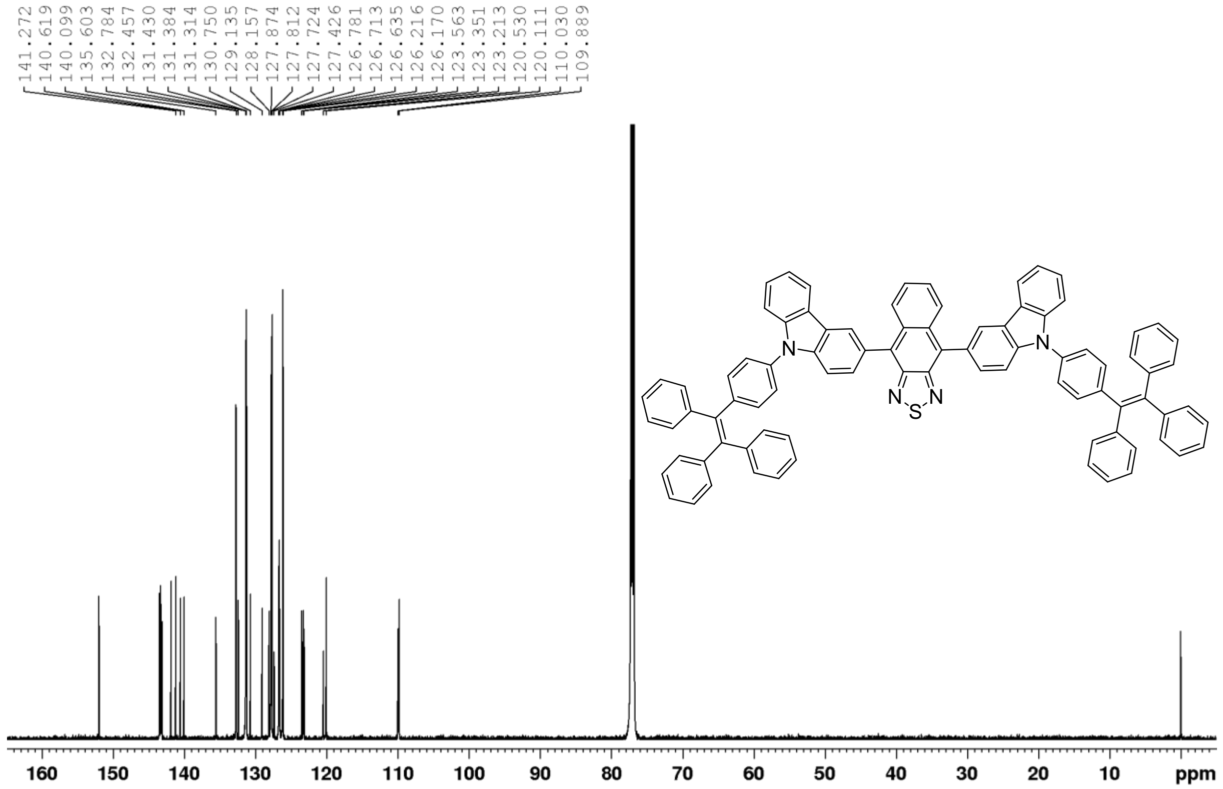

Figure S10: ^{13}C NMR spectrum of **TPECNz** in $CDCl_3$ (151 MHz) @ 25 °C.

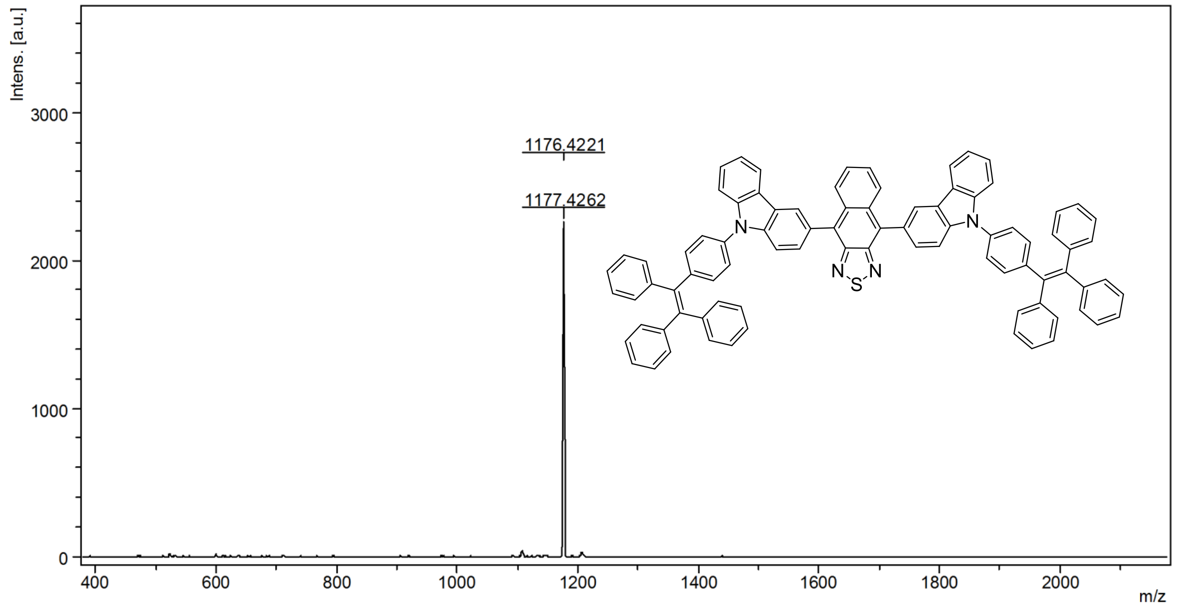

Figure S11: MALDI–TOF mass spectrum $[M]^+$ of **TPECNz**.
